# Validated RP-HPLC based characterization of synergistic antibacterial, antioxidant, and anticancer activities of combined mangosteen pericarp and turmeric extracts

**DOI:** 10.14202/vetworld.2025.2332-2343

**Published:** 2025-08-18

**Authors:** Thippayarat Chahomchuen, Orapin Insuan, Chawanakorn Thavornloha, Nanthiya Hansupalak, Wimonrut Insuan

**Affiliations:** 1 Department of Veterinary Technology, Faculty of Veterinary Technology, Kasetsart University, Bangkok 10900, Thailand; 2 Department of Medical Technology, School of Allied Health Sciences, University of Phayao, Phayao 56000, Thailand; 3 Department of Chemical Engineering, Faculty of Engineering, Kasetsart University, Bangkok 10900, Thailand; 4 Specialized Center of Rubber and Polymer Materials for Agriculture and Industry, Faculty of Science, Kasetsart University, Bangkok 10900, Thailand

**Keywords:** α-mangostin, antibacterial activity, anticancer property, antioxidant activity, curcumin, reversed-phase high-performance liquid chromatography, synergy

## Abstract

**Background and Aim:**

Mangosteen (*Garcinia mangostana*) and turmeric (*Curcuma longa*) are medicinal plants with well-documented antimicrobial, antioxidant, and anticancer properties, attributed to α-mangostin and curcumin, respectively. While their individual bioactivities are recognized, their synergistic potential and standardization through analytical validation remain underexplored, particularly in veterinary and pharmacological applications. This study aimed to (1) evaluate the synergistic antibacterial, antioxidant, and anticancer effects of ethanolic extracts of mangosteen pericarp and turmeric rhizome and (2) develop and validate a high-performance liquid chromatography (HPLC) method for the simultaneous quantification of their major bioactive compounds.

**Materials and Methods:**

Ultrasound-assisted extraction was employed to prepare ethanolic extracts. Antibacterial activities were assessed using disk diffusion, minimum inhibitory concentration (MIC), and minimum bactericidal concentration assays against five bacterial strains, with synergy evaluated through checkerboard fractional inhibitory concentration index. Antioxidant activity was measured by 2,2-diphenyl-1-picrylhydrazyl (DPPH) radical scavenging and total phenolic content (TPC). Cytotoxicity was assessed using 3-(4,5-dimethylthiazol-2-yl)-2,5-diphenyltetrazolium bromide assays on human hepatocellular carcinoma cells (HepG2), human breast adenocarcinoma cells (MCF-7), and human normal fibroblast cells. A reversed-phase HPLC method was developed and validated to simultaneously quantify α-mangostin and curcumin in the combined extract.

**Results:**

Mangosteen and turmeric extracts showed MICs of 3.12 and 31.25μg/mL, respectively. Combined extracts demonstrated additive or synergistic effects against Gram-positive bacteria and enhanced the efficacy of gentamicin (up to 19-fold MIC reduction). The combined extract exhibited the strongest antioxidant activity (half-maximal inhibitory concentration = 5.78 μg/mL) and highest TPC (1227.38 mg gallic acid equivalent/g extract). Cytotoxic assays revealed selective inhibition of HepG2 and MCF-7 cells, with no toxicity toward normal fibroblasts. The validated HPLC method enabled accurate, simultaneous quantification of curcumin (73.23mg/g extract) and α-mangostin (146.80mg/g extract) with excellent linearity (R^2^ > 0.9995) and recovery (99.08%–104.72%).

**Conclusion:**

The combination of mangosteen and turmeric extracts exhibits potent, selective, and synergistic antibacterial and anticancer properties, along with enhanced antioxidant capacity. The validated HPLC method provides a reliable tool for quality control and standardization of such polyherbal formulations, supporting their potential for therapeutic and veterinary applications.

## INTRODUCTION

Natural products, especially those derived from plants, serve as a rich source of biologically active compounds, including primary and secondary meta-bolites. These bioavailable compounds exhibit a wide range of therapeutic effects, benefiting both human and veterinary medicine. Plant-based formulations often consist of single or multiple herbs, resulting in complex and variable chemical compositions influenced by species, cultivation conditions, harvest time, and extraction techniques [1]. Among these, phytochemicals such as alkaloids, flavonoids, terpenoids, and phenolic compounds are particularly valued for their medicinal properties. Notably, crude plant extracts frequently demonstrate higher biological activity than isolated compounds, owing to the synergistic interactions among their constituents [2]. These synergistic eff-ects can enhance therapeutic efficacy, reduce required dosages, improve bioavailability, mitigate toxicity, and minimize adverse effects [2, 3], making them attractive candidates for the development of effective and safer natural therapeutics.

Mangosteen (*Garcinia mangostana*), a tropical plant commonly cultivated in Southeast Asia, including Thailand, Malaysia, and Indonesia, is well known for its medicinal potential [4–6]. Its fruit pericarp is rich in secondary metabolites known as xanthones, a class of polyphenols with potent pharmacological properties. Xanthones exhibit a wide range of bioactivities, including antioxidant [7], antimicrobial [8], anti-inflammatory [9], anticancer [10], and antidiabetic [11] effects. The peel is particularly abundant in α-mangostin, which contributes to its antioxidant, antimicrobial, anti-inflammatory [12], and anticancer [13] properties.

Turmeric (*Curcuma longa*), native to India, Southeast Asia, and Indonesia, is another widely used medicinal plant cultivated across tropical and subtropical regions [14]. It is commonly employed as a natural coloring and flavoring agent in food pro- ducts. Pharmacologically, turmeric is known for its antioxidant, anti-inflammatory, anticancer, antidiabetic, and antimicrobial properties. Its active constituents, curcuminoids (comprising 3%–5% of the dried rhizome), include curcumin, desmethoxycurcu-min, and bisdemethoxycurcumin [15]. Curcumin, in particular, is responsible for a variety of bioactivities, including antioxidant [16], anti-inflammatory [17], anticancer [18], and antibacterial [19] effects.

In recent years, polyherbal formulations, comb-inations of multiple medicinal plants, have gained prominence due to their enhanced therapeutic pote-ntial compared to single-herb preparations [20]. The combination of mangosteen and turmeric has shown promising synergistic effects, enhancing their antibacterial action when used in conjunction with antibiotics [21], as well as their anti-inflammatory [20, 22] and antioxidant [23] properties.

Our previous studies by Insuan *et al*. [24, 25] have also demonstrated that the extraction method significantly influences the biological activity of mangosteen and turmeric, affecting parameters such as antioxidant capacity, total phenolic content (TPC), and antibacterial efficacy.

Despite extensive documentation of the individual therapeutic properties of *G. mangostana* (mangosteen) and *C. longa* (turmeric) – including their antioxidant, antibacterial, anti-inflammatory, and anticancer effects – there remains a paucity of studies evaluating their synergistic interactions in combined formulations. Although a few studies suggest enhanced bioactivity when mangosteen or turmeric are co-administered with conventional antibiotics or other plant extracts, most have not systematically assessed these effects using robust *in vitro* models or standardized analytical methods. Furthermore, the absence of validated analytical techniques for the simultaneous quantification of key bioactive markers (α-mangostin and curcumin) in combined extracts presents a critical limitation in ensuring quality control, reproducibility, and regula-tory compliance for future therapeutic applications. In addition, previous work has largely overlooked comprehensive assessments linking biological efficacy (e.g., minimum inhibitory concentration [MIC], minimum bactericidal concentration [MBC] and half maximal inhibitory concentration [IC_50_] values) with chemical composition (e.g., TPC and high-performance liquid chromatography [HPLC]-validated marker levels), esp-ecially in the context of veterinary and functional food applications.

This study aims to comprehensively evaluate the synergistic biological activities of combined mangost-een pericarp and turmeric rhizome ethanolic extracts, with a focus on antibacterial, antioxidant, and anticancer properties. Specifically, it seeks to:

1. Assess the antimicrobial efficacy of individual and combined extracts against Gram-positive and Gram-negative bacterial strains using MIC, MBC, and checkerboard synergy assays
2. Evaluate antioxidant capacity through DPPH radical scavenging and quantify TPC
3. Investigate the selective cytotoxicity of the extracts against cancerous (human hepatocellular carcinoma cells [HepG2] and human breast adenocarcinoma cells [MCF-7]) and non-cancerous (human normal fibroblast cells [Detroit 551]) cell lines using 3-(4,5-dimethylthiazol-2-yl)-2,5-diphe-nyltetrazolium bromide (MTT) assay
4. Develop and validate a novel reversed-phase HPLC (RP-HPLC) method for the simultaneous qua-ntification of α-mangostin and curcumin in the combined extract, facilitating standardization and quality control.

By integrating bioactivity profiling with validated analytical quantification, this study addresses key methodological and therapeutic gaps and provides a scientific foundation for future applications of man-gosteen–turmeric combinations in complementary medicine and veterinary therapeutics.

## MATERIALS AND METHODS

### Ethical approval

Ethics approval was not required for this study because all experiments were performed *in vitro*.

### Study period and location

The study was conducted from January 2022 to January 2023 at the Laboratory of the Faculty of Veterinary Technology at Kasetsart University and the School of Allied Health Sciences, University of Phayao, Thailand.

### Plant materials and their preparation

Mangosteen pericarp powder and turmeric powder were purchased from an herbal drugstore in Bangkok, Thailand, and stored in a desiccator at room temperature (30°C ±2°C) until use.

### Extraction procedure

An ultrasonic-assisted extraction method (UAE) was employed to prepare extracts from mangosteen pericarp and turmeric. A previous study by Insuan *et al*. [24] indicated that the UAE method, using 70% ethanol, exhibited the highest extraction efficiency for turmeric. In this study, 20 g of each powdered sample was placed in a flask and mixed with ethanol as the extr-action solvent. The extraction process was conducted for 20 min using a rectangular device (Ultrasonic cleaner VGT-1990T, China) at 42 kHz frequency using 240 W input power for 20 min. All experiments were repli- cated at 30°C ±2°C. The ethanolic extract of each plant was then filtered, and the filtrates were evaporated under reduced pressure. The dried resi-dues were weighed and stored at −20°C in amber vials before use.

### Antioxidant activity: DPPH radical scavenging assay

Antioxidant activity was assessed *in vitro* using DPPH radical scavenging activity assays. The capability of extracts from mangosteen, turmeric, and combined extracts to scavenge DPPH radicals was evaluated using a previously established method by Chahomchuen *et al*. [26]. In summary, various concentrations (ranging from 0.7 to 50 μg/mL) of each plant extract and combined extracts dissolved in ethanol were allowed to react with a 0.004% DPPH ethanolic solution. The mixture was thoroughly mixed and then incubated in the dark for 30 min. The absorbance at 517 nm was measured for all extracts. Ascorbic acid, caffeic acid, and Trolox were used as standards for comparison. The inhibition percentage was then calculated using the following equation:

DPPH radical scavenging activity (%) = ([A_control_−A_sample_]/A_control_) × 100%

A_control_ is the absorption of the control reaction (that includes all but the sample reagents) and A_sample_ is the sample absorption (with the DPPH solution). The IC_50_ of plant extracts to inhibit 50% of DPPH was then calculated based on the linear equation of the results with the inhibition percentage against the concentration.

### Quantitation of TPC

The TPC was assessed using the Folin–Ciocalteu reagent, following the method outlined by Insuan *et al*. [24] with some adaptations. In brief, 0.5 mL of crude extract in ethanol was thoroughly mixed with 2.0 mL of diluted Folin–Ciocalteu phenol reagent, followed by the addition of 4 mL of 7.5% (w/v) sodium carbonate. The test tubes were thoroughly vortexed and incubated in darkness at 30°C ±2°C for 30 min. After 30 min, the absorbance of the reaction mixture was measured at 765 nm using an ultraviolet (UV)-visible spectrophotometer (Genesys 20, Thermo Scientific, USA). A standard calibration curve of gallic acid was prepared, and the TPC of the extract samples was determined by extrapolating this curve (y = 0.0689x + 0.0361; R^2^ = 0.9952).

### Development and validation of HPLC methods

#### HPLC analysis

The major components of the mangosteen and turmeric extracts were analyzed by HPLC using a Waters e2695 separation module (Waters, USA). The column used was C18 (150 × 4.6 mm). A 10 μL volume of each sample solution was injected, and elution was conducted using a gradient elution system at a flow rate of 1 mL/min at 40°C, following the method outlined by Insuan *et al*. [24] with a modification to the mobile phase ratio (changed from 50:50 v/v acetonitrile and 0.1% ortho-phosphoric acid). The mobile phase comprised acetonitrile (A) and 0.1% phosphoric acid (B), programmed as follows: 40%–50% A (0–5 min), 50% A (5–8 min), 50%–70% A (8–10 min), and 70%–95% A (10–20 min). The absorption of the analyzed and quantified compounds was investigated using a UV detector at wavelengths of 254, 320, and 427 nm. The RP-HPLC method was validated for the simulta-neous quantification of two bioactive compounds in combined extracts: α-mangostin in mangosteen and curcumin in turmeric. Standard solutions of α-mangost in and curcumin were prepared in methanol, aliquoted, and stored at −20°C for future analysis. The crude extracts were filtered through 0.45 μm PTFE syringe and properly diluted to the required concentration for subsequent analysis. The α-mangostin and curcumin contents of the crude extracts were identified based on their retention times and by comparison with standards under the same conditions.

#### Validation of the HPLC Method

Validation parameters, including linearity, limit of determination (LOD), limit of quantification (LOQ), precision, and accuracy, were assessed to validate the developed method. Calibration curves of α-mangostin and curcumin were constructed by plotting the peak area against the concentration and regression equations were determined accordingly.

### Testing of antimicrobial activity

#### Bacterial strains and cultures used

The five reference strains used in this study included *Staphylococcus aureus* (American Type Culture Collection [ATCC] 25923), *Staphylococcus epidermidis* (ATCC 29663), *Staphylococcus intermedius* (ATCC 29663), *Escherichia coli* (ATCC 25922), and *Pseudomo-nas aeruginosa* (ATCC 27853). These strains were stored at −80°C in nutrient broth (NB) (Difco, USA) with 20% glycerol. Nutrient Agar (Difco, USA) was used to cul-tivate a single colony of each bacterium. All bacterial suspensions were prepared from fresh cultures grown overnight in NB at 37°C under agitation conditions. All antimicrobial assays were performed under biosafety level 2 to ensure safety and prevent contamination.

#### Disk diffusion assay

The disk diffusion method was used to assess the antibacterial activities of the turmeric and mangosteen extracts. Microbial cell suspensions were adjusted to 0.5 McFarland turbidity standards, aiming for approximately 10^8^ colony-forming unit (CFU)/mL in Mueller–Hinton broth (MHB) (Difco, USA), which was validated by performing serial dilutions and CFU enumeration. Subsequently, the tested bacteria were spread evenly onto the surface of Mueller–Hinton agar (MHA) (Difco, USA) in 9-cm diameter Petri dishes (Alpha plus, China) using sterile cotton swabs. The agar plates were left to air dry. Sterile filter paper disks with a diameter of 6 mm (Whatman, England) saturated with 10 μL of various test extracts (10 mg/mL in dimethyl sulfoxide [DMSO]) were then aseptically placed on the agar surface. Plates were incubated at 37°C for 16–18 h, after which the inhibition zone diameters were measured in mm. Gentamicin solution, prepared in DMSO with 2 μg/disk as the antibiotic standard, was used as a positive control. Negative controls consisted of 10 μL of DMSO.

#### MIC and MBC determination

The MIC and MBC of each plant extract were determined using the micro-dilution method according to Clinical Laboratory Standards Institute (CLSI) [27], with slight modifications. Serial double dilutions of the extracts and antibiotics were prepared in MHB and transferred into 96-well microtiter plates at final concentrations of 1.95–500 μg/mL, 1.56–400 μg/mL, and 0.0078–2.00 μg/mL for turmeric extract, mangosteen extract, and gentamicin, respectively. To each well, 100 μL of each dilution of the extracts or gentamicin and 100 μL of bacterial suspension in MHB at a concentration of 1 × 10^6^ CFU/mL were added to a final bacterial number of approximately 1.5 × 10^5^ CFU per well. Two controls were included: a sterility control with MHB medium alone and a positive control with MHB medium without extracts or antibiotics to control inoculum viability. DMSO (final volume of 5% v/v) was added as a negative control. Plates were incubated for 24 h at 37°C, and the OD was measured at 595 nm using a spectrophotometer (iMark, Bio-Rad, U.S.A.). The MIC was defined as the lowest concentration that resulted in ≥90% reduction in turbidity compared with the untreated control. In addition, bacterial viability was confirmed by adding a resazurin solution (TCI, Japan). After incubation for 30 min at 37°C, color alteration was observed in wells containing viable bacteria. MIC was determined. Ten microliters of an aqueous MIC and the more concentrated test dilution were inoculated into MHA, and the MBC values were determined after overnight incubation at 37°C. The tests were performed in triplicate.

#### Checkerboard synergy assay

The checkerboard titration method following the method outlined by Omokhua *et al*. [28] was used to evaluate the synergistic effects of turmeric, mangosteen, and gentamicin. The concentration ranges used were 3.90–500 μg/mL for turmeric, 0.39–50 μg/mL for mangosteen, and 0.0078–1.00 μg/mL for gentamicin. Briefly, one extract was diluted two-fold in MHB along the vertical rows of a 96-well microtiter plate, while another extract was cross-diluted horizontally by two-fold serial dilution. Bacterial suspension was added to each well to obtain a final concentration of 1.5 × 10^5^ CFU/mL. Plates were then incubated at 37°C for 24 h. After adding resazurin solution and incubating at 37°C for 30 min, the MIC was defined as the lowest concentration that exhibited complete growth inhibition and showed no color change. The tests were performed in triplicate.

The synergistic effects were evaluated using the fractional inhibition concentration (FIC). The FIC values were calculated as follows:

FIC = MIC in combination/MIC alone.

To assess the interaction between agents, the fractional inhibitory concentration index (FICI) was determined using the following formula:

FICI = (MIC of A in the combination/MIC of A alone) + (MIC of B in the combination/MIC of B alone).

FICI has been interpreted as per CLSI guidelines as follows: synergistic effect (FICI ≤ 0.5), partially syn-ergistic (0.5 < FICI ≤ 0.75), additive effect (0.76 ≤ FICI ≤ 1), indifference (1 ≤ FICI ≤ 4), and antagonistic effect (FICI > 4) [29].

### Cell culture and cytotoxicity assay

HepG2, MCF-7, and Detroit 551 were obtained from the ATCC (USA). Cells were cultured in Dulbecco’s Modified Eagle’s Medium (Gibco; USA) supplemented with 10% fetal bovine serum, 100 U/mL penicillin, and 100 mg/mL streptomycin under a humidified (95%) atmosphere of 5% CO_2_ at 37°C.

The cytotoxicity of the plant extracts (turmeric, mangosteen, and combined turmeric and mangosteen extracts) was determined using an MTT tetrazolium reduction assay [30]. Two cancerous cell lines (HepG2 and MCF-7) and a control serving non-cancerous Detroit 551 cells were selected for this study. The plant extracts were prepared in DMSO at a final concen-tration of 0.1% (v/v). HepG2 (1 × 10^4^ cells/well), MCF-7 (1 × 10^4^ cells/well), or Detroit 551 (5 × 10^3^ cells/well) cells was seeded in 96-well plates overnight and then treated with different concentrations of the plant extracts (1.5625–100 μg/mL) for 24, 48, and 72 h, res-pectively. Next, the cells were incubated with MTT solution at a final concentration of 0.5 mg/mL for 4 h at 37°C. Then, the supernatant was removed, 100 μL of DMSO was added to dissolve the formazan crystals, and the absorbance was determined at 540 nm using a microplate spectrophotometer (Berthold Technologies GmbH & Co. KG; Germany). Untreated cells with a cell viability of 100% were used as controls. The cell viability of the treated wells was calculated as a percentage relative to that of the control. The IC_50_ values of the plant extracts on various cell lines were calculated, and the results are expressed as mean ± standard deviation (SD).

### Statistical analysis

The experimental results are reported as the mean ± SD of triplicate assays. Statistical analysis was performed using Microsoft Excel 365 software (Microsoft Corp., Washington, USA). A one-way analysis of variance was used to determine statistical significance (p < 0.05).

## RESULTS

### Antioxidant activity

The antioxidant activity and TPC of turmeric and mangosteen extracts were assessed, as shown in Table 1. The DPPH radical scavenging assay revealed that the mixed extract (1:1 v/v) exhibited the highest antioxidant activity (5.78 ± 0.05 μg/mL), surpassing mangosteen (9.36 ± 0.14 μg/mL) and turmeric (14.19 ± 0.55 μg/mL) extracts.

**Table 1:** Antioxidant activities and total phenolic content of mangosteen and turmeric extract (n = 3).

Extract	2,2-diphenyl-1- picrylhydrazyl (IC_50_, µg/mL)	Total phenolic content (mg GAE/g extract)
Mangosteen extract	9.36 ± 0.14^c^	400.10 ± 18.10^a^
Turmeric extract	14.19 ± 0.55^d^	409.77 ± 36.20^a^
Mixed extract	5.78 ± 0.05^b^	1227.38 ± 67.38^b^
Ascorbic acid	2.25 ± 0.02^a^	-
Trolox	5.42 ± 0.12^b^	-
Caffeic acid	2.60 ± 0.10^a^	-

Different lowercase superscripts (a, b, c, and d) within column indicate a significant difference (p < 0.05) among various extracts

IC_50_=Half maximal inhibitory concentration, GAE=Gallic acid equivalent

### TPC

TPC analysis (Table 1) showed that the mixed extract contained 1227.38 ± 67.38 mg gallic acid equivalent (GAE)/g dried extract, which was higher than the TPC contents of mangosteen (400.10 ± 18.10 mg GAE/g dried extract) and turmeric (409.77 ± 36.20 mg GAE/g dried extract) extracts. A strong correlation (−0.826) was observed between antioxidant activity and TPC.

### Quantification of α-mangostin and curcumin in crude extracts

**Figure 1:**
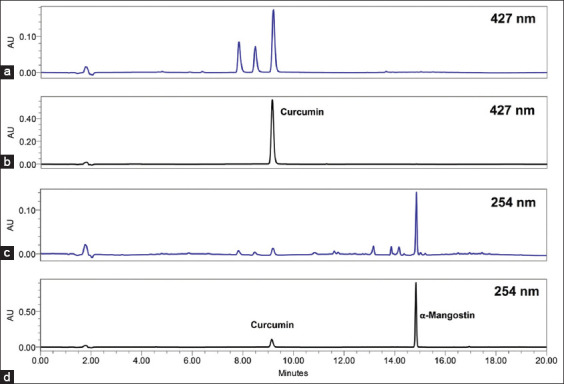
Simultaneous separation of curcumin and α-mangostin through high-performance liquid chromatography. Chromatogram of sample mixture (a) and standard mixture (b) at 427 and sample mixture (c) and standard mixture (d) at 254 nm, flow rate of 1.0 mL/min for 20 min.

Based on the HPLC analysis, the major compounds of mangosteen and turmeric, as well as the sample mixture, were tentatively identified by comparison with the standards. The HPLC method was developed to select the best chromatographic parameters. Figure 1 shows the representative chromatograms obtained from the HPLC analysis.

Quantitative validation of curcumin in turmeric and α-mangostin in mangosteen was performed using gradient elution mode. The results in Table 2 demon-strate good linearity, with regression equations of y = 90150x − 26729 for curcumin and y = 38693x − 37153 for α-mangostin. The LOD and LOQ for curcumin were 0.009 and 0.031 μg/mL, while for α-mangostin, they were 0.014 and 0.047 μg/mL, respectively.

**Table 2:** Method validation parameters for the quantification of curcumin and α-mangostin by HPLC method.

Parameter	Curcumin	α-Mangostin
Scanning wavelength (nm)	427	254
Linear range (µg/mL)	0.7–200	1.5–300
Regression equation	y=90150x−26729	y=38693x−37153
Regression coefficient	R^2^=0.9996	R^2^=0.9995
Slope ± SD^a^	90149.66 ± 282.23	38590 ± 180.39
LOD (µg/mL)	0.0093	0.0140
LOQ (µg/mL)	0.0313	0.0467
Intraday precision (%RSD)^a^		
Retention time	0.019	0.016
Peak area	0.387	0.142
Interday precision (%RSD)^a^		
Retention time	0.452	0.072
Peak area	1.066	5.635
Accuracy (%recovery±SD)^a^		
5 (µg/mL)	100.07 ± 1.08	104.52 ± 0.85
50 (µg/mL)	99.08 ± 2.77	104.46 ± 3.17
100 (µg/mL)	99.79 ± 2.84	104.72 ± 2.65

a Average of three determinations, LOD=Limit of detection, LOQ=Limit of quantification, RSD=Relative standard deviation, SD=Standard deviation, HPLC=High-performance liquid chromatography

The intraday precision results for curcumin and α-mangostin were found to be 0.019 and 0.016 for retention time and 0.387 and 0.142 for peak area, respectively. The interday precision results had %RSD values lower than 5%. The accuracy was confirmed based on the average percentage recovery at three different levels: 99.64% for curcumin and 104.56% for α-mangostin.

The developed HPLC method was used for the quantification of the combined extracts, and the results revealed that curcumin and α-mangostin were found to be 73.23 ± 1.79 and 146.80 ± 9.37 mg/g extract (Table 3), respectively, at retention times of 9.17 and 14.83 min.

**Table 3:** RP-HPLC quantification of curcumin and α-mangostin in combined extract (n=3).

Sample	Standard marker	Retention time (minute)	Concentration (mg/g extract)^a^
Mixed extract	Curcumin	9.17	73.23 ± 1.79
	α-mangostin	14.83	146.80 ± 9.37

a Data are expressed as mean ± standard deviation, RP-HPLC=Reversed-phase high-performance liquid chromatography

### Antibacterial activity of turmeric and mangosteen extracts

**Figure 2:**
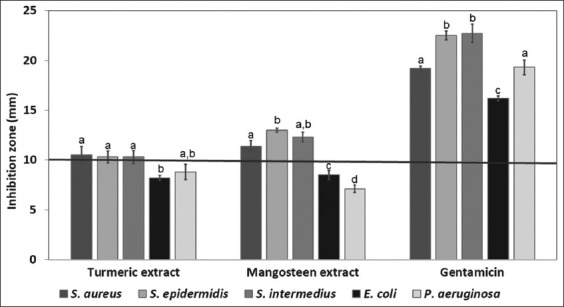
The screening of the antimicrobial activity of turmeric extract, mangosteen extract, and gentamicin (the diameter of the inhibition zone, including 6 mm of paper disk, is reported as mean (n = 3) ± standard deviation). Different lowercase superscripts (a, b, c, d) within the same group are significantly different (p < 0.05).

The extracts were screened for antimicrobial act-ivity using the disk diffusion assay, and the inhibition zone diameters are presented in Figure 2. Turmeric and mangosteen extracts demonstrated antibacterial activity against all tested bacteria. Remarkably, these extracts exhibited stronger inhibition of Gram-positive bacteria (*S. aureus*, *S. epidermidis*, and *S. intermedius*) than Gram-negative bacteria (*E. coli* and *P. aeruginosa*).

In this study, inhibition zone sizes larger than 10 mm were considered significant indicators of strain susceptibility, allowing for further investigation of their potent inhibitory effect. The MICs of the extracts were determined using a broth microdilution assay. As shown in Table 4, the turmeric and mangosteen extracts exerted significant inhibitory effects against *S. aureus*, *S. epidermidis*, and *S. intermedius* with equal MIC values at 31.25 μg/mL and 3.12 μg/mL, respectively.

**Table 4:** MIC and MBC values and MBC: MIC ratios of turmeric and mangosteen extract against each tested bacterium.

Bacterial strain	Turmeric			Mangosteen			Gentamicin		
		
	MIC (µg/mL)	MBC (µg/mL)	MBC/MIC ratio	MIC (µg/mL)	MBC (µg/mL)	MBC/MIC ratio	MIC (µg/mL)	MBC (µg/mL)	MBC/MIC ratio
*Staphylococcus aureus*	31.25	62.50	2	3.12	6.25	2	0.06	0.12	2
*Staphylococcus epidermidis*	31.25	62.50	2	3.12	6.25	2	0.12	0.25	2
*Staphylococcus intermedius*	31.25	31.25	1	3.12	3.12	1	0.03	0.12	4

MBC=Minimum bactericidal concentration, MIC=Minimum inhibitory concentration

The MBC of turmeric extract ranged from 31.25 to 62.50 μg/mL against all tested strains. The MBC of the mangosteen extract ranged from 3.12 to 6.25 μg/mL for all tested strains. Mangosteen and turmeric extracts demonstrated potent bactericidal activity against all tested bacterial strains, as evidenced by MBC/MIC ratios consistently ranging from 1 to 2.

### Synergistic effect of turmeric and mangosteen extracts in combination

Table 5 shows the MICs and FICI of the extracts and gentamicin in combination. It was examined that the combination of turmeric and mangosteen extracts had varying effects depending on the bacterial strain. It was observed that the combination of *S. epidermidis* and *S. intermedius* resulted in indifference, indicating that the combined effect was not significantly different from that of using the extracts individually.

**Table 5:** MIC and FICI values of turmeric and mangosteen extracts and gentamicin in combination.

Test sample	Bacterial strain	*Staphylococcus aureus*	*Staphylococcus epidermidis*	*Staphylococcus intermedius*
Turmeric/Mangosteen	MIC combined (µg/mL)	15.62/1.56	31.25/1.56	31.25/3.125
	ΣFIC	0.50/0.49	1.0/0.49	1.0/1.0
	FICI/Outcome	0.99/Additive	1.49/Indifferent	2.00/Indifferent
Turmeric/Gentamicin	MIC combined (µg/mL)	7.81/0.0125	7.81/0.0062	15.6/0.0125
	ΣFIC	0.25/0.20	0.25/0.05	0.49/0.40
	FICI/Outcome	0.45/Synergy	0.30/Synergy	0.90/Additive
Mangosteen/Gentamicin	MIC combined (µg/mL)	1.56/0.025	3.12/0.025	1.56/0.0125
	ΣFIC	0.49/0.40	1.0/0.20	0.49/0.40
	FICI/Outcome	0.89/Additive	1.20/Indifferent	0.89/Additive

FIC index: <0.5 synergy, 0.5–0.75 partial synergy, 0.76–1.0 additive effect, 1–4 indifferent, >4 antagonism

MIC=Minimum inhibitory concentration, FICI=Fractional inhibitory concentration index

However, for *S. aureus*, the combination reduced the MIC by 2-fold relative to the individual extracts, sugg-esting an additive effect. The combination of turmeric extract with gentamicin produced a markedly stronger antimicrobial effect, showing a synergistic interaction (FICI ≤ 0.5) against *S. aureus* and *S. epidermidis*. Com-pared with gentamicin alone, this combination reduced the required antibiotic dose by approximately 4.8-fold and 19-fold, respectively, indicating significantly enhanced efficacy compared with either agent used independently.

These findings highlight the potential of this combination to improve antibacterial outcomes beyond the sum of individual effects. However, against *S. intermedius*, the turmeric–gentamicin combination exhibited an additive effect with a 2.4-fold reduction in the MIC of gentamicin, suggesting improved antibac-terial activity. Similarly, the combination of mangosteen extract with gentamicin exhibited an additive effect against *S. aureus* and *S. intermedius*, resulting in a 2.4-fold reduction in the MIC of gentamicin; however, it showed an indifferent effect against *S. epidermidis*.

### Cytotoxic effects of turmeric and mangosteen extracts

**Figure 3:**
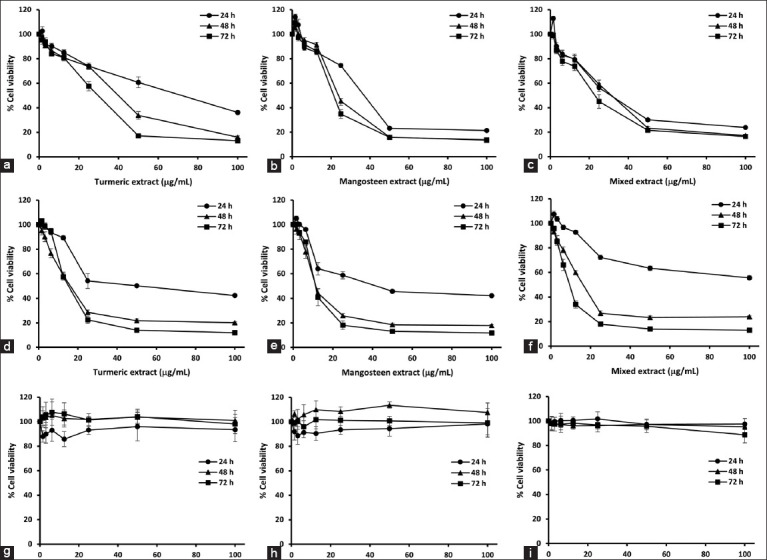
The viability of HepG2 (a-c), MCF-7 (d-f), and Detroit 551 (g-i) cells after treatment with various concentrations of turmeric extract, mangosteen extract, and mixed turmeric and mangosteen extract, respectively, at 24, 48, and 72 h. The data are shown as means ± standard deviation of three independent experiments. HepG2=Human hepatocellular carcinoma cells, MCF-7=Human breast adenocarcinoma cells, Detroit 551=Human normal fibroblast cells.

The cytotoxicity of turmeric and mangosteen extracts against HepG2, MCF-7, and Detroit 551 is represented in Figure 3, and IC_50_ values are shown in Table 6. In this study, Detroit 551 cells were used as normal cells for comparison with cancer-derived cell models.

**Table 6:** IC_50_ values of turmeric, mangosteen, and mixed turmeric and mangosteen extracts on HepG2, MCF-7, and Detroit 551 cells at 24, 48, and 72 h detected by MTT assay.

Extract	IC_50_ value (µg/mL)		

	HepG2	MCF-7	Detroit 551
Turmeric extract			
24 h	78.71 ± 6.24	54.75 ± 5.62	>100
48 h	40.46 ± 3.21	16.94 ± 2.09	>100
72 h	30.70 ± 0.25	15.88 ± 2.11	>100
Mangosteen extract			
24 h	35.27 ± 1.39	29.46 ± 7.72	>100
48 h	27.25 ± 1.21	11.92 ± 3.37	>100
72 h	28.54 ± 1.11	12.93 ± 4.91	>100
Mixed extract			
24 h	34.84 ± 2.48	>100	>100
48 h	31.98 ± 1.45	16.17 ± 0.74	>100
72 h	30.27 ± 2.80	9.02 ± 2.35	>100

Data expressed as mean ± standard deviation of three independent experiments

IC_50_=Half maximal inhibitory concentration, HepG2=Human hepatocellular carcinoma cells, MCF-7=Human breast adenocarcinoma cells, Detroit 551=Human normal fibroblast cells, MTT=3-(4,5-dimethylthiazol-2-yl)-2,5-diphenyltetrazolium bromide

Based on the results, turmeric, mangosteen, and the combined turmeric and mangosteen extracts had no cytotoxic effects on normal cells (% cell survival > 80%), with IC_50_ values more than 100 μg/mL. In contrast, treatment with the extracts produced toxic effects on HepG2 and MCF-7 cells.

All treatments produced growth inhibition for both tumor cell types that were both dose dependent (1.5625–100 μg/mL) and time dependent (24, 48, and 72 h). At 24 h, the IC_50_ values of the turmeric extract against HepG2 and MCF-7 cells were 78.71 and 54.75 μg/mL, respectively. When the incubation period was extended to 48 h, the HepG2 and MCF-7 cells had IC_50_ values of 40.46 and 16.94 μg/mL, respectively. At 72 h, the IC_50_ values of the HepG2 and MCF-7 cells were 30.70 and 15.88 μg/mL, respectively.

Similarly, the mangosteen extract exerted a cytotoxic effect on both HepG2 and MCF-7 tumor cell lines at 24 h, with IC_50_ values of 35.27 and 29.46 μg/mL, respectively. HepG2 and MCF-7 proliferation was also inhibited after treatment with the mangosteen extract for 48 h. Furthermore, the mangosteen extract had a high cytotoxic effect on HepG2 and MCF-7, with IC_50_ values of 28.54 and 12.93 μg/mL, respectively, at 72 h.

For the combined turmeric and mangosteen extract, the IC_50_ value for the HepG2 cells was 34.84 μg/mL after 24 h incubation, 31.98 μg/mL at 48 h, and 30.27 μg/mL at 72 h. There was lower cytotoxicity to MCF-7, with a high IC_50_ value that was >100 μg/mL at 24 h. However, the IC_50_ values of the MCF-7 cells were 16.17 and 9.02 μg/mL after the incubation was extended to 48 and 72 h, respectively.

These findings suggest that extracts from turmeric and mangosteen exhibit cytotoxic effects against cancer cells while remaining non-toxic to normal cells.

## DISCUSSION

### Analytical performance of the validated HPLC method

The validated HPLC method developed in this study demonstrates high precision, sensitivity, and reproducibility for the simultaneous quantification of curcumin and α-mangostin. Compared with previous methods by Lee *et al*. [31], Muchtaridi *et al*. [32], and Setyaningsih *et al*. [33], our gradient RP-HPLC method provides better separation efficiency and a relatively short analysis time (20 min). Setyaningsih *et al*. [33] reported that the isocratic RP-HPLC method can be used for quantitative determination of curcumin in extracts from *C. longa*, with a total analysis time of 10 min. Similarly, Lee *et al*. [31] demonstrated that a gradient RP-HPLC method using a C18 column (250 × 4.6 mm) and a mobile phase of 0.1% formic acid (v/v) and acetonitrile could determine curcumin within 15 min.

In contrast, the methodology of Muchtaridi *et al*. [32] for determining α-mangostin in mangosteen extract differed significantly in column dimensions, elution mode (isocratic), and total runtime. Few studies have reported an HPLC method capable of quantifying both curcumin and α-mangostin simultaneously in a mixed herbal extract. In our work, we developed and validated a single, efficient gradient RP-HPLC method for this purpose – providing a novel and essential tool for quality control in polyherbal formulations derived from two different species.

### Synergistic antioxidant properties and phenolic content

The results confirmed that the mixed extract had enhanced antioxidant activity, likely due to synergistic effects between turmeric and mangosteen constituents. The observed strong correlation (r = −0.826) between TPC and antioxidant activity suggests that polyphenols significantly contribute to free radical scavenging.

This correlation supports the role of phenolic compounds as primary contributors to antioxidant capacity. Furthermore, the combination of turmeric and mangosteen outperformed individual extracts in terms of antioxidant efficiency, confirming the beneficial impact of synergistic interaction. These findings are consistent with previous reports indicating that phenolic compounds, including flavonoids, curcuminoids, and xanthones, exhibit potent antioxidant effects through mechanisms such as hydrogen atom donation and radi-cal stabilization [34].

### Antibacterial efficacy and antibiotic synergy

The antibacterial efficacy of turmeric and man-gosteen extracts against Gram-positive bacteria aligns with previous studies [5, 19, 35–37]. The observed bactericidal activity is likely attributed to active compounds such as curcumin and α-mangostin, which are known to disrupt bacterial membrane integrity and increase permeability [38, 39].

The significant reduction in MIC values indicates that these extracts enhance the efficacy of antibiotics. The antibacterial effect was further confirmed by MBC/MIC ratios ranging from 1 to 2, well below the threshold of ≤4 used to define bactericidal action [40].

Furthermore, the synergistic effects observed when these extracts were combined with gentamicin offer a promising strategy for combating antibiotic resistance. These combinations may serve as effective antibiotic adjuvants by enhancing cytoplasmic antibiotic access and amplifying antimicrobial activity [41].

### Selective cytotoxicity and anticancer potential

Cytotoxicity analysis revealed that turmeric and mangosteen extracts selectively inhibited cancer cells without affecting normal fibroblasts, positio-ning them as promising candidates for anticancer applications. Their dose- and time-dependent effects on HepG2 and MCF-7 cells were consistent with previously reported anticancer mechanisms of curcumin and α-mangostin [42, 43].

The extracts demonstrated strong cytotoxic eff-ects against both tumor cell lines while maintaining high viability in normal cells, suggesting a favorable therapeutic index.

### Overall implications and therapeutic potential

Overall, this study provides valuable insights into the pharmacological potential of turmeric and mangosteen extracts. The validated HPLC method enables the accurate and simultaneous quantifica-tion of curcumin and α-mangostin in mixed extracts. In parallel, the observed potent antioxidant, antibact-erial, and anticancer activities, combined with selec-tive cytotoxicity and synergistic efficacy, support the therapeutic relevance of these plant-derived com-pounds. Together, these findings underline the utility of turmeric and mangosteen in the development of multifunctional herbal therapeutics.

## CONCLUSION

This study demonstrated the enhanced phar-macological potential of combined *G. mangostana* (mangosteen) and *C. longa* (turmeric) extracts, high-lighting their synergistic antioxidant, antibacterial, and anticancer activities. The mixed extract exhibited superior antioxidant capacity (IC_50_ = 5.78 ± 0.05 μg/mL) compared to individual extracts, which correlated strongly with its high TPC (1227.38 ± 67.38 mg GAE/g extract), underscoring the central role of polyphenols in free radical scavenging. Quantitative RP-HPLC analysis revealed significant concentrations of cur- cumin (73.23 ± 1.79 mg/g extract) and α-mangostin (146.80 ± 9.37 mg/g extract), with validated pre- cision, sensitivity, and recovery rates (99.64% for curcumin and 104.56% for α-mangostin), enabling accurate quality control of the combined extract.

The extracts exhibited notable bactericidal acti-vity (MBC/MIC ratios of 1–2) against Gram-positive pathogens, particularly *S. aureus*, *S. epidermidis*, and *S. intermedius*, with MIC values of 31.25 μg/mL (turmeric) and 3.12 μg/mL (mangosteen). Synergistic interactions with gentamicin enhanced antimicrobial efficacy up to 19-fold, suggesting practical potential as an antibiotic adjuvant for managing drug-resistant infections. In addition, the extracts selectively inhibited HepG2 and MCF-7 cancer cell lines in a dose- and time-dependent manner, with no toxicity observed in normal fibroblasts (Detroit 551), indicating their safety and relevance in cancer therapy.

The findings provide a promising foundation for developing dual-extract phytopharmaceuticals or veterinary therapeutics with multifunctional bio-activity. The validated HPLC method serves as a valuable tool for standardizing combined botanical products – critical for clinical translation and regulatory approval.

This study’s strengths include the validation of an efficient and reproducible RP-HPLC method optim-ized for the simultaneous quantification of curcumin and α-mangostin in a polyherbal formulation. It also provides comprehensive bioactivity profiling using antioxidant, antimicrobial, and cytotoxicity assays and offers evidence of synergistic effects with antibiotics and selective cytotoxicity toward cancer cells while preserving normal cell viability. These outcomes increase the translational relevance of the findings. However, the study is limited to *in vitro* models, and the results may not be fully applicable to *in vivo* conditions. In addition, only a limited panel of bacterial strains and cancer cell lines was exa- mined, which may restrict broader applicability.

Future studies should include *in vivo* models to validate pharmacokinetics, bioavailability, and safety profiles. Mechanistic investigations at the molecular level, including gene and protein expression, would help elucidate the underlying pathways of action. Moreover, formulating these extracts into advanced delivery systems such as nanoparticles may further optimize their therapeutic efficacy.

In conclusion, the combined extract of turmeric and mangosteen exhibits synergistic therapeutic potential with significant antioxidant, antibacterial, and anticancer effects. The validated HPLC method serves as a critical analytical tool for ensuring quality assurance. These findings lay the groundwork for further development of this polyherbal formulation as a safe, effective, and multifunctional natural therapeutic candidate for integrative medicine.

## AUTHORS’ CONTRIBUTIONS

TC: Conceptualization, methodology, antimicro-bial testing, writing – original draft, and writing – review and editing. OI: Conceptualization, methodology, investigation of cell viability testing, writing – original draft, and writing – review and editing. CT: Investigation of antioxidant activity. NH: Conceptualization WI: Conceptualization, methodology, extraction, determination of anti-oxidant activity and markers content, writing –original draft, and writing – review and editing. All authors have read and approved the final manuscript.
